# Development of a rapid and sensitive immunochromatographic strip based on EuNPs-ES fluorescent probe for the detection of early *Trichinella spiralis*-specific IgG antibody in pigs

**DOI:** 10.1186/s13567-021-00951-9

**Published:** 2021-06-11

**Authors:** Xinyu Wang, Bin Tang, Ying Zhao, Jing Ding, Nan Wang, Yan Liu, Zijian Dong, Xiangdong Sun, Quangang Xu, Mingyuan Liu, Xiaolei Liu

**Affiliations:** 1 Key Laboratory of Zoonosis Research, Ministry of Education, Institute of Zoonosis, College of Veterinary Medicine, Jilin University, Changchun, 130062 China; 2 Department of Nephrology, First Hospital of Jilin University, Changchun, 130021 China; 3 Jiangsu Co-Innovation Center for Prevention and Control of Important Animal Infectious Diseases and Zoonoses, Yangzhou, Jiangsu China; 4 College of Veterinary Medicine, College of Animal Science and Technology, Jilin Agricultural University, Changchun, China; 5 China Animal Health and Epidemiology Center, Qingdao, Shandong China

**Keywords:** *Trichinella spiralis*, Excretory–secretory, Eu (III) nanoparticles, Immunochromatographic strip, Serodiagnosis

## Abstract

Trichinellosis, which is caused by nematodes of the genus *Trichinella*, is one of the most important zoonotic parasite diseases in the world. A rapid and sensitive immunochromatographic strip (ICS) based on Eu (III) nanoparticles (EuNPs) was developed for the detection of *Trichinella spiralis* (*T. spiralis*) infection in pigs. *T. spiralis* muscle larvae excretory secretory or preadult worm excretory secretory (ML-ES or PAW-ES) antigens were conjugated with EuNPs probes to capture *T. spiralis*-specific antibodies in pig sera, after which the complex bound to mouse anti-pig IgG deposited on the test line (T-line), producing a fluorescent signal. In the pigs infected with 100, 1000 and 10 000 ML, seroconversion was first detectable for the EuNPs-ML-ES ICS at 30, 25 and 21 days post-infection (dpi) and for the EuNPs-PAW-ES ICS at 25, 21 and 17 dpi. These results show that EuNPs-PAW-ES ICS detects anti-*Trichinella* IgG in pigs 4–5 days earlier that test using ML-ES antigens. Our ICS have no cross reaction with other parasite infection sera. Furthermore, the detection process could be completed in 10 min. This study indicated that our ICS can be used for the detection of the circulating antibodies in early *T. spiralis* infection and provide a novel method for on-site detection of *T. spiralis* infection in pigs*.*

**Supplementary Information:**

The online version contains supplementary material available at 10.1186/s13567-021-00951-9.

## Introduction

Trichinellosis is a parasitic zoonotic disease caused by nematodes of the genus *Trichinella*, which have a global geographical distribution and affects a wide variety of more than 150 carnivorous and omnivorous animals [1–3]. Most of human infections are caused by *Trichinella spiralis* (*T*. *spiralis*) by consuming raw or uncooked meat containing infective muscle larvae (ML), and humans usually present with clinical symptoms [3]. According to a recent report by the International Commission on Trichinellosis (ICT), the number of humans infected with *Trichinella* spp. was 65 818 from 1986 to 2009 [4]. Meanwhile, 38 794 cases of *T. spiralis* infection were reported in China from 1964 to 2011, of which 336 led to death [5–7]. From 2000 to 2015, 2251 people were diagnosed as trichinellosis in Yunnan Province, China [8]. Therefore, China has become a region greatly affected by trichinellosis in recent decades. In view of the above situation, China has spent 2.2 billion CNY each year on preventing and detecting trichinellosis [5]. Moreover, pigs are the most common vector for transmitting trichinellosis, which is primarily caused by *T. spiralis* infection in China [9]. Therefore, to ensure food safety and maintain the economic stability of the breeding pig industry, there is an urgent need to develop a method of on-site detection of *T. spiralis* in pigs.

After the host ingests the meat containing *T. spiralis*, the infective larvae are released in the muscle tissue of the ingested meat and migrate into the small intestine. Then, they shed four molts and develop into adult worms (AW) within 30–36 h post-infection. Four days later, female adult worms release newborn larvae (NBL) which arrive the host muscle tissue through the circulation of blood and lymph fluid and parasitize in muscle cell for years. Muscle larvae excretory secretory (ML-ES) antigens are produced by *T. spiralis* ML, and ML-ES antigens are commonly used in serological methods for the diagnosis of *Trichinella* infections in animals and humans [10–12]. However, ML-ES antigens are phase-specific and can cause false negatives in the 3^rd^–4^th^ weeks post-infection [13]. The false negatives are due to the lag time of the immune response following the ingestion of infective larvae. Therefore, the ELISA or immunochromatographic strip (ICS) methods based on ML-ES antigens cannot be recommended for the detection of early *T. spiralis* infections. Previous study reported that adult worm excretory secretory (AW-ES) can be identified by the anti-*Trichinella* antibodies in early *T. spiralis* infection [14, 15]. Meanwhile, preadult worm (PAW) obtained at 6 h post-infection makes early contact with the host immune system compared with AW [16, 17]. Therefore, ML-ES and PAW-ES antigens were applied and evaluated for the detection of *T. spiralis* infection by ICS in this study.

The diagnosis of *Trichinella* infection utilizes direct and indirect methods, and the gold standard method is the artificial digestion method that is recommended by the World Organization for Animal Health (OIE). The ELISA detection method based on ML-ES antigens is a widely accepted serological method for monitoring *Trichinella* infection and investigating its epidemiology [18, 19]. However, the above described methods have the disadvantage of long detection time, laborious and expensive equipment. With the development of immunochromatography technology, ICS methods that utilize gold nanoparticles, up-converting phosphor nanoparticles, magnetic particles and quantum dots have been widely used in testing for pregnancy and chemical residues [20–22]. Moreover, colloidal gold nanoparticles as labels on ICS have been used to detect *T. spiralis* infection [23, 24]. Recently, EuNPs, which are time-resolved fluorescent microspheres with the advantages of a large Stokes shift, a narrow emission spectrum and a long quenching time, have begun to replace colloidal gold as the labels used in mainstream ICS methods for clinical disease detection, such as hepatitis B virus and porcine epidemic diarrhea virus [25, 26]. For investigating the seroconversion of infected pigs, a novel EuNPs-ICS detection method based on ML-ES antigens and PAW-ES antigens was developed for *T. spiralis* infection.

In this study, an EuNPs-ES fluorescent probe conjugated with ML-ES and PAW-ES antigens were established for detecting *T. spiralis*-specific IgG antibodies in pigs by ICS, respectively. The sensitivity and specificity of two kinds of ICS were investigated, which will help promoting the on-site diagnosis of *Trichinella* infection. To date, there has no reports on detecting *T. spiralis* infection by fluorescent probe-based ICS.

## Materials and methods

### Reagents and instruments

Bovine serum albumin (BSA) and Tween-20 were obtained from Solarbio (Beijing, China). EDC was obtained from TCI (Shanghai, China). COOH-modified europium nanoparticles (EuNPs) were obtained from Thermo (USA). Mouse anti-pig monoclonal IgG antibodies and rabbit anti-goat IgG antibodies were obtained from Beijing Biolab Technology (Beijing, China). Goat anti-rabbit IgG was obtained from Cell Signal Technology (USA). NC membranes (Millipore 135) and Ultra-15 3 kDa centrifugal filters were obtained from Millipore (USA). The sample pads, absorbent paper and plastic backing were obtained from Jinbiao Biotech (Shanghai, China). BCA kits were obtained from Beyotime Biotechnology (Shanghai, China). The ES commercial ELISA kit was obtained from Qiagen (Germany).

XYZ3060 dispenser was obtained from Biodot (USA). A TRF fluorescence quantitative analyzer was obtained from Weice Biotech (Nanjing, China). A UV lamp was obtained from Shenzhen Feike Technology (Shenzhen, China).

### Preparation of ES antigens

The collection of ML-ES antigens followed the previous methods and procedures [27, 28]. A total of 10 specific pathogen-free (SPF) SD rats were orally inoculated with 3500 *T. spiralis* ML (T1, ISS534) and were euthanized at 35 dpi, and tissues were used for the recovery of ML by the artificial digestion method. After washing three times with 0.9% saline solution, the ML were cultured in serum-free RPMI-1640 medium containing antibiotics (90 U/mL penicillin and 90 μg/mL streptomycin) at 37 °C for 18 h in 5% CO_2_. After separation by centrifugation at 1000 × *g*, the ML culture supernatant was concentrated by Ultra-15 3 kDa centrifugal filters and the protein concentration was measured by BCA kits.

The collection of PAW-ES antigens followed the previous methods and procedures [14]. After infection with 10 000 *T. spiralis* ML, the SD rats were euthanized at 6 h after infection. The entire small intestine was removed from the abdominal cavity and soaked in 0.9% saline solution at 37 °C for 2 h (with 180 U/mL penicillin and 180 μg/mL streptomycin). The culture and collection of PAW-ES antigens were performed as ML-ES antigens.

Finally, ML-ES antigens and PAW-ES antigens were analyzed by SDS-PAGE (Additional file 1).

### Pigs and serum samples

The experiment of this part has been done by our laboratory before [27]. Fifteen Large White pigs were divided into three groups (five pigs per group) and inoculated with 100, 1000 and 10 000 *T. spiralis* ML (T1, ISS534). Serum samples were collected from pigs at 0, 7, 9, 11, 13, 15, 17, 19, 21, 25, 30, 35, 45, 60, 90 and 120 dpi. Finally, all pigs were sacrificed to calculate larvae per gram of muscle (lpg) at 120 dpi. These results were showed in supplement material, which indicated all pigs were successfully infected with *T. spiralis* ML (Additional file 4).

One hundred serum samples were collected from 25 Large White pigs infected with 400 *T. spiralis* ML at 25, 30, 35, 45 dpi. Another 170 serum samples were collected from 170 parasite-free Large White pigs as negative controls to calculate the cut-off value. A total of 38 serum samples were collected from pigs infected with *Clonorchis sinensis* (2), *Cryptosporidium parvum* (2), *Taenia sodium* (2), *Toxoplasma gondii* (2)*, Ascaris suum* (15), *Trichuris suis* (5) and *Metastrongylus elongatus* (10)*.* All pigs were healthy according to the Chinese Laboratory General Requirements for Animal Experiments. Before the experiment, all pigs underwent a week-long healthy observation and were examined for parasite eggs in feces and blood by the flotation and sedimentation method. Furthermore, all pigs were fed basic diet without adding antibiotics and were kept under standard pig houses in our laboratory under the care of a professional breeder.

### Preparation of fluorescent probe

EuNPs were conjugated with ES antigens as follows [29]: firstly, 10 μL of EuNPs were centrifuged at 14 000×*g* for 15 min to remove glycerol and phosphate, and then 10 µL of 1 mg/mL EDC, 10 µL of 1 mg/mL NHS, 100 µL of MES and EuNPs were mixed and stirred for 45 min to activate the EuNPs completely at room temperature. After removing the unbound EDC and NHS by centrifugation at 14 000 × *g* for 15 min, 20 µg the ML-ES and 20 µg PAW-ES antigens were mixed into the solution, and incubated for 3 h. Then, 200 µL of 5% BSA was added to the mixtures to block the unreacted active sites overnight at 4 °C. Finally, the EuNPs-ML-ES and EuNPs-PAW-ES fluorescent probes were resuspended in 200 µL of preservation buffer (50 mm/L PBS containing 1% BSA and 1% ProClin). The EuNPs-goat anti-rabbit IgG fluorescent probe was also prepared by the same procedure described above.

To identify the change of fluorescent probe after coupling, the morphology of the fluorescent probe was observed by transmission electron microscopy (TEM) and the absorption peak of the fluorescent probe was detected by fluorescent microplate reader.

### Preparation of the ICS

EuNPs-conjugated ML-ES or PAW-ES antigens were designed as probes to capture anti-*T. spiralis* antibodies, and conjugated goat anti-rabbit IgG antibodies were used as an indicator probe. Mouse anti-pig monoclonal IgG and rabbit anti-goat IgG were immobilized on the NC membrane as the test line (T-line) and control line (C-line), respectively. Using the XYZ3060 dispenser at a rate of 0.8 μL/cm, mouse anti-pig monoclonal IgG (1 mg/mL) and rabbit anti-goat IgG (1 mg/mL) were applied to the NC membrane as the T-line and C-line, respectively. Finally, NC membranes absorbed paper and sample pad were assembled into strips (3.8 mm-wide).

For detection, 100 µL the running buffer (0.9% NaCl, 1.5% BSA, 0.05% Tween-20), 1 µL serum sample, 1 µL EuNPs-ES and 0.5 µL EuNPs-goat anti-rabbit IgG were mixed in a bioclean tube. Then, after the sample was added into the ICS, the ICS was placed into a 37 °C incubator for 10 min. The fluorescent signal of the T-line was observed under a UV lamp at 365 nm wavelength and was analyzed by a TRF reader.

### The cut-off value for the ICS and Qiagen ELISA

A total of 170 serum samples from 170 parasite-free pigs were detected by ICS, and the result of ICS were analyzed by a TRF reader. The cut-off value was calculated by $\overline{{\text{x}}}$  ± 2 SD of the T-line fluorescence values of the 170 serum samples. A T-line values for a sample below the cut-off value was judged as negative, and a value above or equal to the cut-off value was judged as positive. The cut off value of Qiagen ELISA follow the manufacturer’s instructions: S/*P* values = (OD_Sample_ − OD_Negative Control_)/(OD_Positive Control_ − OD_Negative Control_).

### Cross-reactivity with other parasites

To evaluate the specificity of ICS, a total of 38 serum samples from pigs infected with *Clonorchis sinensis*, *Cryptosporidium parvum*, *Taenia sodium*, *Toxoplasma gondii*, *Ascaris suum*, *Trichuris suis* and *Metastrongylus elongatus* were detected by the EuNPs-ML-ES ICS and EuNPs-PAW-ES ICS.

### Seroconversion of infected pigs detected by ICS and Qiagen ELISA

Serum samples from pigs infected with 100, 1000 and 10 000 ML (five animals per group) at 0, 7, 9, 11, 13, 15, 17, 19, 21, 25, 30, 35, 45, 60, 90 and 120 dpi were detected by EuNPs-ML-ES ICS, EuNPs-PAW-ES ICS and Qiagen ELISA. The test results for the middle- and high-dose ELISA groups have been published previously, and the results for the low-dose group are shown in Additional file 2 [30].

A standard indirect ELISA protocol was performed by Qiagen ELISA kit. Briefly, 90 µL of sample diluent and 10 µL pre-diluted serum were added into each sample well, then wells were incubated for 60 min at room temperature (18–25 °C). After removing solution by aspiration, wells were rinsed each well of Wash Buffer. 100 µL ready-to-use Conjugation was added into each well and incubated for 30 min at room temperature. After removing solution and wells were rinsed again. 100 µL TMB Substrate Solution was added into each well and incubated for 10 min at room temperature. Stop the reaction by adding 100 µL Stop Solution per well, and optical density (OD) values was measured by plate reader at 450 nm.

### The sensitivity and specificity of EuNPs-ML-ES ICS and EuNPs-PAW-ES ICS

To investigate the sensitivity and specificity of ICS, 100 serum samples were collected from 25 Large White pigs infected with 400 *T. spiralis* ML at 25, 30, 35, 45 dpi, at the end of experiment, all pigs were confirmed to be infected with *T. spiralis* ML by artificial digestion method. 170 serum samples collected from 170 parasite-free pigs as negative, which also confirm by artificial digestion method. These serum samples were detected by EuNPs-ML-ES ICS and EuNPs-PAW-ES ICS.

### Clinical application of ICS

To validate the method in the field in an endemic area where *Trichinella* circulates among the backyard pigs. We collected 1032 pork and 1032 serum samples from slaughterhouses and rural areas in Inner Mongolia, Sichuan and Guangxi provinces in China where *T. spiralis* infection were suspected. All pork samples were detected by artificial digestion method, and serum sample were detected by the two ICS.

### Statistical analysis

After the results of ICS were analyzed by fluorescent reader, the T-line fluorescence values were imported into GraphPad Prism8 software. SPSS 19 was used for statistical analysis, Chi-square tests were performed on ICS with standard artificial digestion.

## Results

### The process of immunochromatography

**Figure 1:**
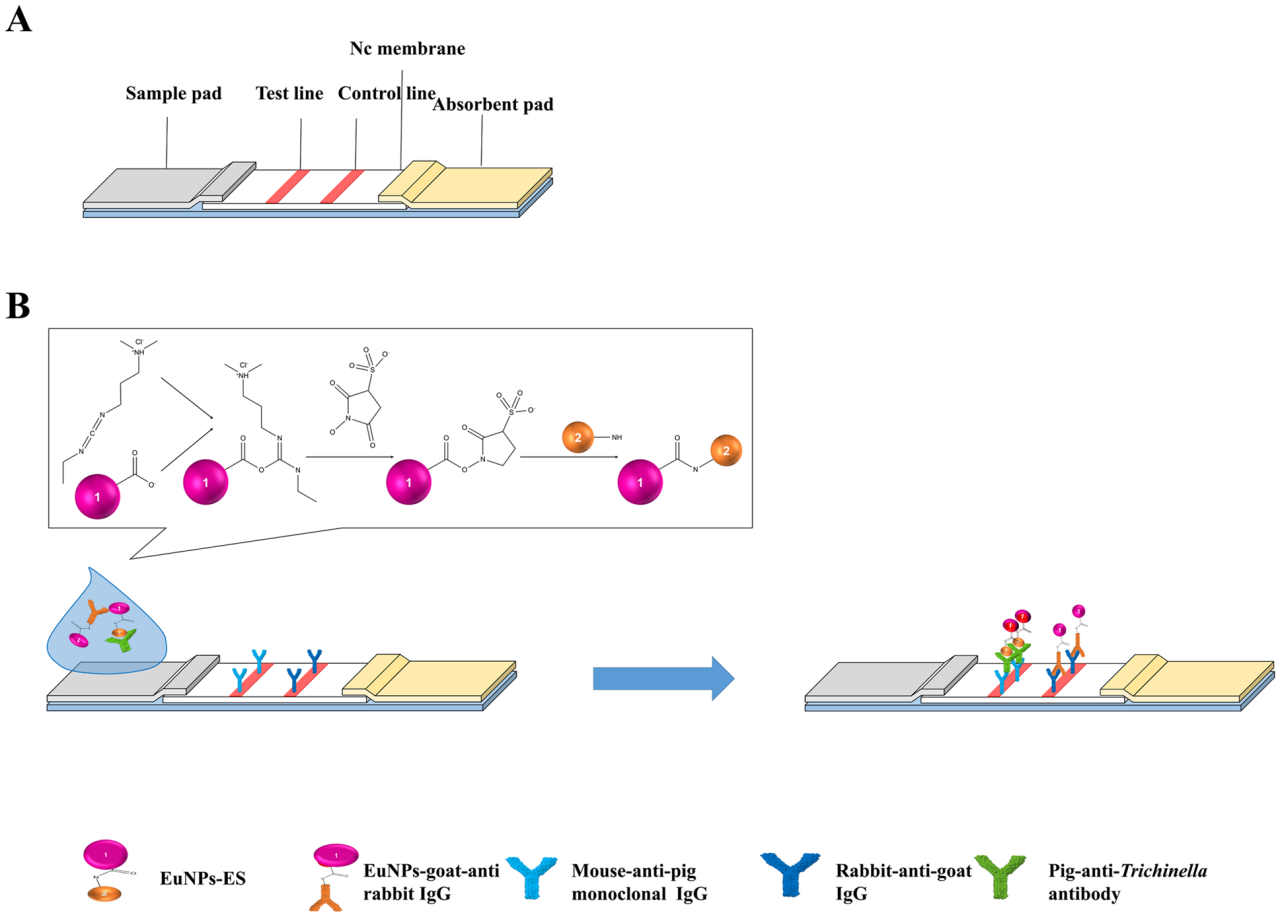
**The schematic of EuNPs-ICS.** **A** The composition of immunochromatographic strips. **B** The process of immunochromatographic strips.

The ICS used for detecting anti-*T. spiralis* antibodies is based on a classic indirect method. As shown in Figure 1A, when a positive serum sample was detected, the EuNPs-ES probe captured the anti-*T. spiralis* antibodies. Then, under the traction of the absorption pad, the mixture migrated across the NC membrane and reacted with the mouse anti-pig monoclonal IgG antibodies on the T-line. Meanwhile, the EuNPs-goat anti-rabbit IgG antibodies probe reacted with the rabbit anti-goat IgG antibodies on the C-line. When the negative sample was detected, only the EuNPs-goat anti-rabbit IgG probe was captured on the C-line (Figure 1B).

### The diagnosis of EuNPs-ICS

**Figure 2:**
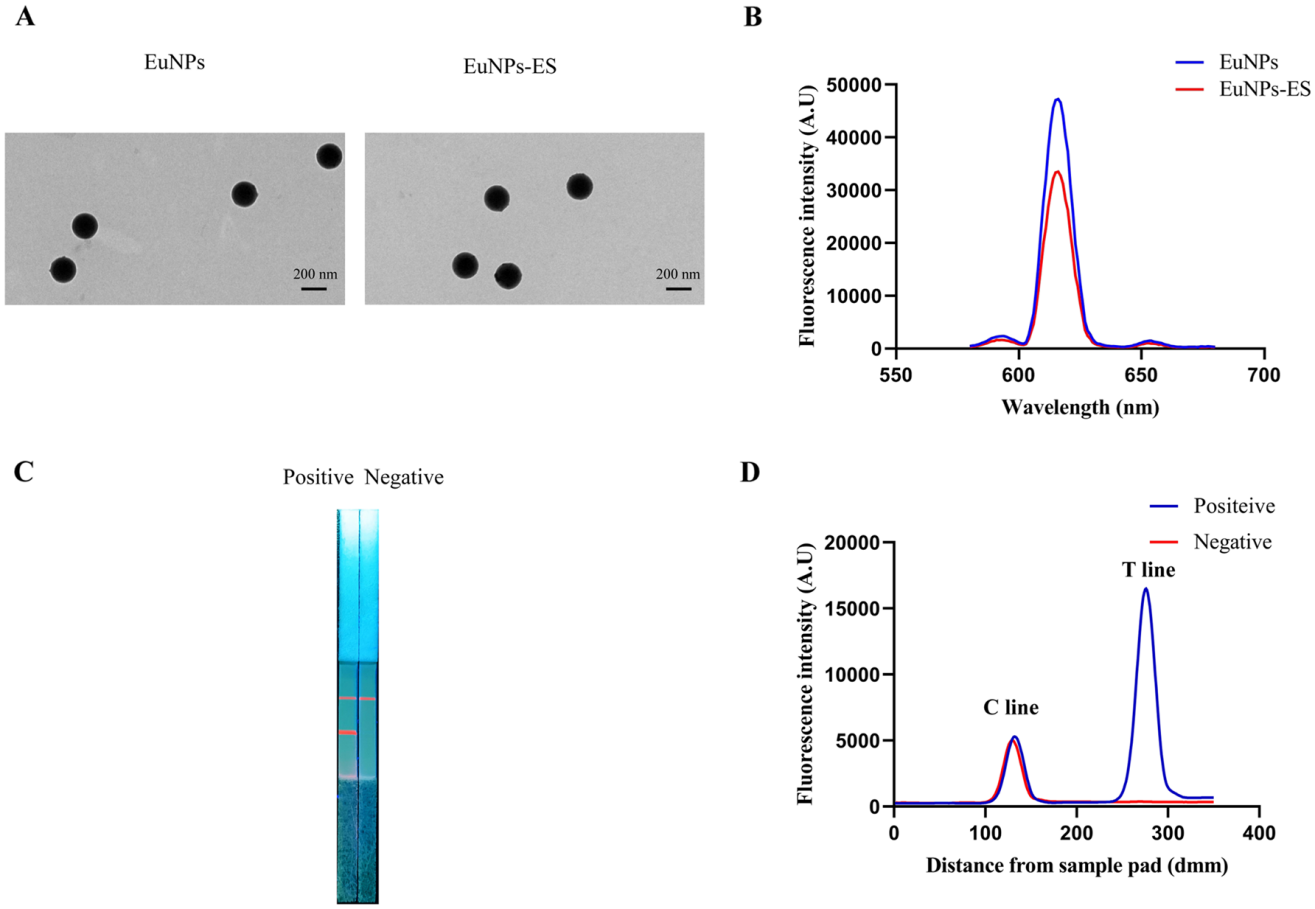
**The diagnosis of EuNPs-ICS.** **A** The image of EuNPs or EuNPs-ES probe by TEM. **B** The maximal emission peak of EuNPs or EuNPs-ES probe. **C** The image of positive and negative serum samples detected by ICS under UV lamp. **D** Fluorescence peak heights readout curve of ICS for detecting positive and negative serum samples.

The EuNPs probe was imaged by TEM (Figure 2A). It is clear that the two probes have a uniform circular shape and almost the same diameter. The maximal emission peak appeared in 625 nm and there was no difference between EuNPs and EuNPs-ES probe (Figure 2B). When serum samples were detected by the ICS, a strong fluorescence signal appeared on the T-line for the positive serum sample, but no fluorescence signal was found on the T-line for the negative serum sample. In addition, regardless of whether positive or negative serum sample was detected, the C-line showed a certain degree of fluorescence, which indicated that the ICS could distinguish negative and positive serum sample (Figures 2C and D).

### The cut-off value of the ICS

Normal 170 serum samples from parasite-free pigs were detected by the ICS. Based on the means ± 2SD value, the cut-off value for the EuNPs-ML-ES ICS and the EuNPs-PAW-ES ICS were 115.71 and 112.97, respectively. Interestingly, the cut off value of EuNPs-ML-ES ICS is slightly high than EuNPs-PAW-ES ICS.

Moreover, the T-line fluorescence values for 170 serum samples were imported into GraphPad Prism8 software, and we found that the data conformed to a normal distribution (Figure 5).

### Cross-reactivity with other parasites serum samples

**Figure 3:**
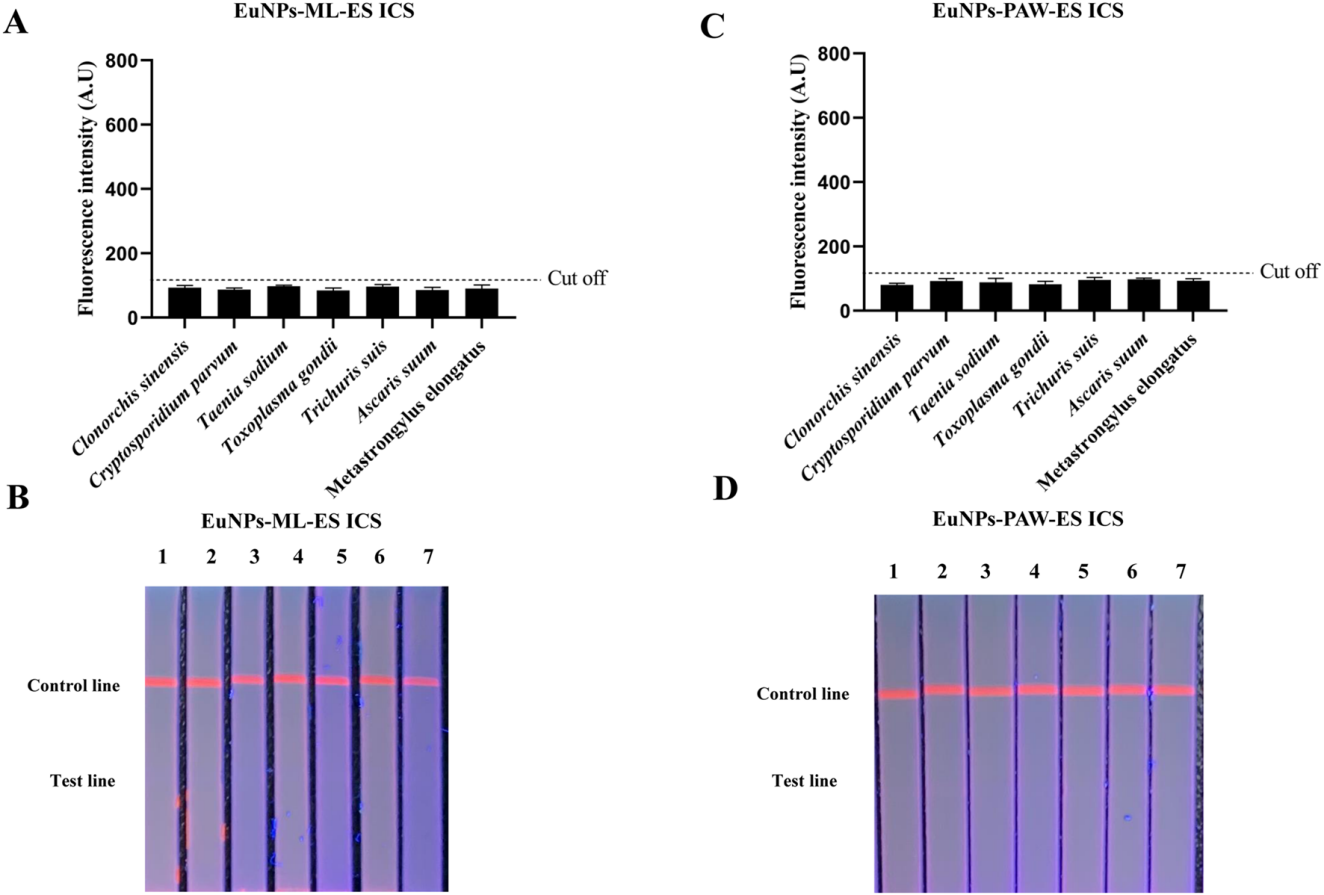
**The specificity of ICS.** In total of 38 serum samples from pigs infected with *Clonorchis sinensis, Cryptosporidium parvum*, *Toxoplasma gondii*, *Taenia sodium*, *Ascaris suum*, *Trichuris suis* and *Meatstrongylus elongatus* were detected by EuNPs-ML-ES ICS and EuNPs-PAW-ES ICS. **A**, **C** T line fluorescence values of ICS analyzed by TRF reader. **B**, **D** Visual results of ICS under ultra-violet light (1, *Clonorchis sinensis* 2, *Cryptosporidium parvum* 3, *Taenia sodium* 4, *Toxoplasma gondii* 5, *Trichuris suis* 6, *Ascaris suum* 7, *Meatstrongylus elongatus*).

The T-line fluorescence values for serum samples from other parasites were below the cut-off value, and no positive bands appeared in ICS for other parasites serum samples, which indicated that the EuNPs-ICS has no cross-reactivity with other parasites serum samples (Figure 3).

### Seroconversion of infected pigs detected by ICS and Qiagen ELISA

**Figure 4:**
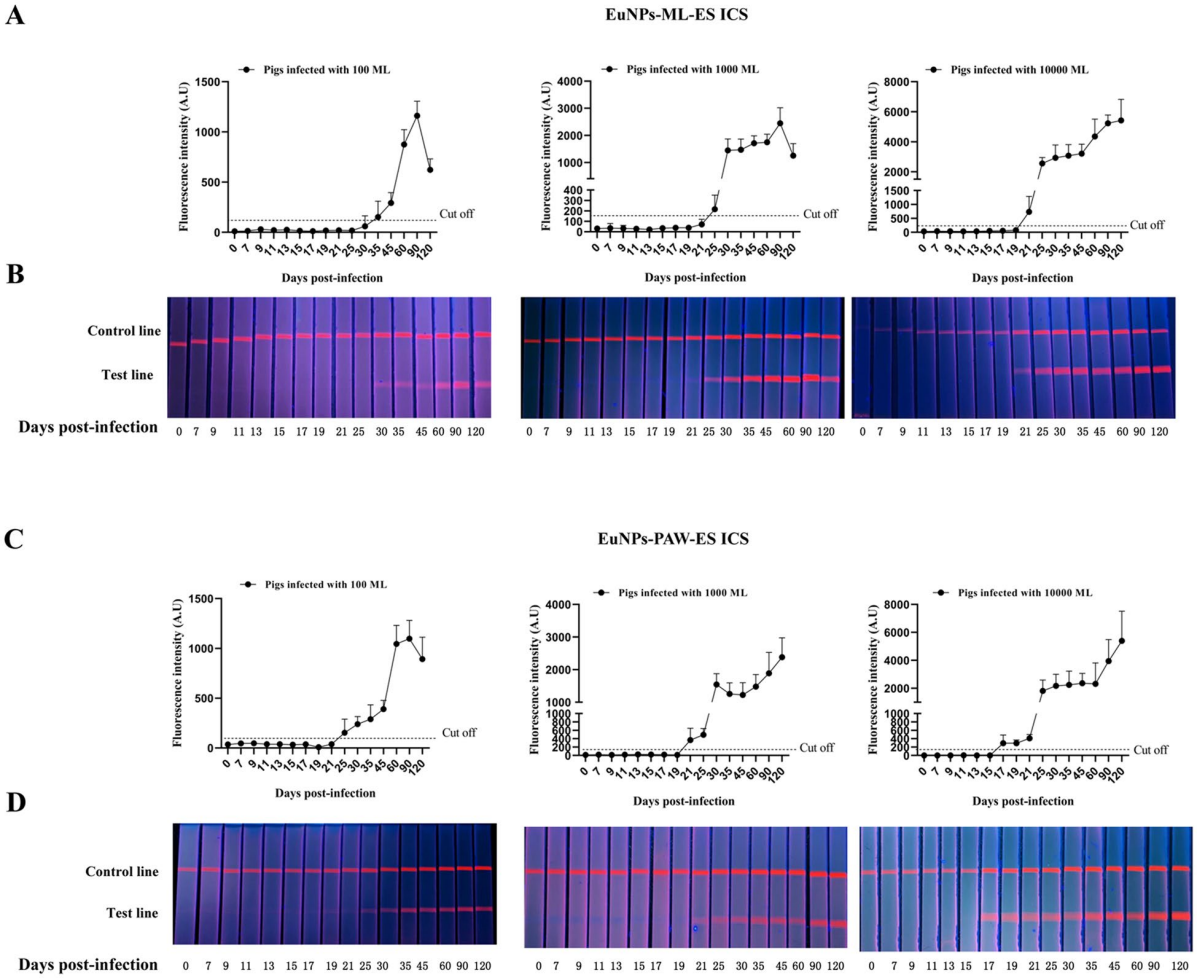
**Seroconversion of infected pigs detected by ICS.** Serum samples from pigs infected with 100, 1000 and 10 000 ML at 0, 7, 9, 11, 13, 15, 17, 19, 21, 25, 30, 35, 45, 60, 90, and 120 dpi were detected by EuNPs-ML-ES ICS and EuNPs-PAW-ES ICS. **A**, **C** T-line fluorescence values analyzed by TRF reader shown as means ± SD of five independent pigs per doses. **B**, **D** Visual results under ultra-violet light for one pig per doses.

As shown in Figure 4, the fluorescence intensity of the T-line increased as the number of dpi or ML infection doses increased. Based on the cut-off value, in the pigs infected with 100, 1000 and 10 000 ML, seroconversion was first detectable by the EuNPs-ML-ES ICS at 30, 25 and 21 dpi and by the EuNPs-PAW-ES ICS at 25, 21 and 17 dpi, respectively (Figure 4). In the pigs infected with 100, 1000 and 10 000 ML, 100% antibody positivity was detected by the EuNPs-ML-ES ICS at 45, 30 and 25 dpi and by the EuNPs-PAW-ES ICS at 35, 25 and 19 dpi (Table 1). When serum samples were detected by Qiagen ELISA, in pigs infected with 100, 1000 and 10 000 ML, seroconversion was first detectable at 30, 25, and 21 dpi, and 100% antibody positivity was detected at 45, 30 and 25 dpi (Table 1). These results indicated seroconversion of infection pigs was detected by EuNPs-PAW ICS earlier than EuNPs-ML-ES ICS and Qiagen ELISA.

**Table 1:** **Seroconversion time of infected pigs detected by ICS and Qiagen ELISA**

Dpi			
ICS/doses^a^	100	1000	10 000
EuNPs-ML-ES	30,30,35,45,45	25,25,30,30,30	21,21,21,21,25
EuNPs-PAW-ES	25,25,30,30,35	21,21,25,25,25	17,17,19,19,19
Qiagen ELISA	30,30,30,35,45	25,25,25,30,30	21,21,21,21,25

dpi: days post-infection.

doses^a^: larval inoculation dose in pigs.

### The sensitivity and specificity of EuNPs-ML-ES ICS and EuNPs-PAW-ES ICS

**Figure 5:**
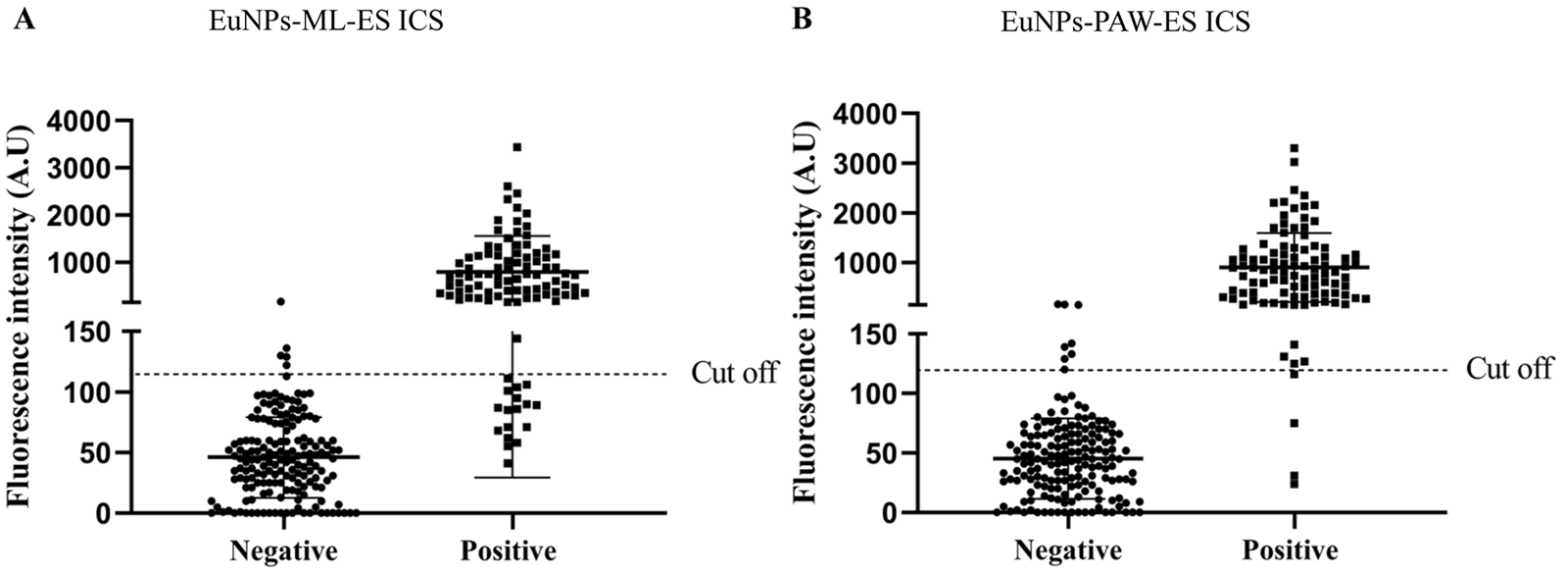
**Distribution of results from EuNPs-ML-ES ICS and EuNPs-PAW-ES ICS.** **A** Distribution of negative serum samples and positive serum samples detected by EuNPs-ML-ES ICS. **B** Distribution of negative serum samples and positive serum samples detected by EuNPs-PAW-ES ICS.

The sensitivity of the EuNPs-ML-ES ICS and EuNPs-PAW-ES ICS was 85% and 97%, respectively. The specificity of the EuNPs-ML-ES ICS and EuNPs-PAW-ES ICS was 96.47% and 95.29%, respectively (Figure 5). The Chi-square test of golden standard artificial digestion and EuNPs-PAW-ES ICS gave a *P* value of 0.227 (McNemar χ^2^, *P* > 0.05), and Kappa values of 0.917. The Chi-square test of golden standard artificial digestion and EuNPs-ML-ES ICS gave a *P* value of 0.078 (McNemar χ^2^, *P* > 0.05), and Kappa values of 0.83 (Table 2). Compared with EuNPs-ML-ES ICS, EuNPs-PAW-ES ICS could distinguish precisely positive and negative sera and showed a strong agreement with the artificial digestion methods.

**Table 2:** **Comparison of EuNPs-PAW-ES ICS and EuNPs-ML-ES ICS with standard artificial digestion**

	Artificial digestion		Total	Sensitivity (%)	Specificity (%)	Mc Nemar χ^2^	Kappa
	+	−					
*EuNPs-PAW-ES ICS*							
+	97	8	270	97	95.29	*P* > 0.05 (0.227)	0.917
−	3	162					
*EuNPs-ML-ES ICS*							
+	85	6	270	85	96.47	*P* > 0.05 (0.078)	0.83
−	15	164					

### Clinical application of ICS

For investigating the performance of clinical application, artificial digestion and ICS method were evaluated by detection of clinical samples. As shown in Table 3, In total of 1032 serum samples and 1032 pork samples, three serum samples were detected as antibody positive by ICS, one pork sample was detected as positive by artificial digestion (Additional file 3, Table 3).

**Table 3:** **Clinical application of ICS with artificial digestion**

Method/results	Positive	Negative	In total of
Artificial digestion	1	1031	1032
EuNPs-ML ICS	3	1029	1032
EuNPs-PAW ICS	3	1029	1032

## Discussion

Established a rapid and sensitive detection method for *T. spiralis* infection is a priority topic. Due to the advantages of a large stokes shift, a narrow emission spectrum and a long quenching time, the EuNPs-ICS shows high sensitivity and specificity compared to the colloidal gold ICS and is widely used in disease diagnosis, food safety, public health and environmental protection [31, 32]. However, EuNPs-ICS has not been applied in detection of *T. spiralis* infection. In this study, a novel and rapid EuNPs-ICS was developed for investigating seroconversion of infected pigs, and the specificity and sensitivity of ICS were evaluated.

ES antigens are secreted by the cuticle surface and stichosome of *T. spiralis* [33]. Tyvelose, the component of the ES antigens, is also found in Gram-negative bacterial lipopolysaccharides and *Ascaris* eggs, so the serological method established by using the ES antigens has cross-reactivity with sera from infection with other parasites [34]. To improve the specificity of ICS, anti-pig polyclonal IgG antibodies were replaced with mouse anti-pig monoclonal IgG antibodies as the T-line capture antibodies. To enhance the blocking effect, different concentrations of BSA was added into coupling system and loading buffer system. Our results indicate that our ICS have high specificity and have no cross reaction with sera from infection with other parasites.

ELISA method based on ML-ES antigens is commonly used in diagnosis of *T. spiralis* infection. But ML-ES antigens are phase-specific, the false negative results were often obtained by ELISA method on detection of early *T. spiralis* infection [13]. In this study, PAW-ES and ML-ES antigens were applied in EuNPs-ICS to investigate the seroconversion of infection pigs compared with Qiagen ELISA. The limit detection of EuNPs-ML-ES ICS was consistent with Qiagen ELISA, whereas the limit detection of EuNPs-PAW-ES ICS was lower than Qiagen ELISA, suggesting that PAW-ES antigens are more sensitive to antibodies produced in early *T. spiralis* infection. Moreover, our previous study showed that *T. spiralis* infection first be detectable at 17 dpi by standard artificial digestion [27]. EuNPs-PAW-ES ICS gives a high sensitivity and specificity and has a strong agreement with standard artificial digestion in this study, which confirmed EuNPs-PAW-ES ICS has the advantages in early serodiagnosis of *T. spiralis* infection. Furthermore, it had been previously reported that the early antigens of *T. spiralis* contain early serodiagnostic markers for *Trichinella* infection and could be recognized by the early infection sera [15]. Our results also indicated PAW-ES antigens applied in ICS can detect *T. spiralis* infection earlier than ML-ES antigens, and it shortened effectively the window periods for detection of *T. spiralis* infection. It maybe because the the PAW-ES antigens were exposed firstly to the host’s systematic immune system which triggers the generation of *Trichinella*-specifc antibodies during the intestinal stage of *T. spiralis* infection [35, 36].

Although colloidal gold ICS based on ML-ES antigens has been established for detection of *T. spiralis* infection [23, 24], fluorescent probe-based ICS as a new method was introduced in detection of *T. spiralis* infection, and ML-ES antigens and PAW-ES antigens were evaluated by ICS. Compared with the colloidal gold ICS, the EuNPs-ICS could not only distinguish the negative and positive serum by the naked eye observation, but also judge the results more precisely by the fluorescence reader. Seroconversion was firstly detected by colloidal gold ICS at 28, 21 and 18 dpi for 200, 2000 and 20 000 *T. spiralis* ML [23], and detected by EuNPs-PAW-ES ICS at 25, 21 and 17 dpi for 100, 1000 and 10 000 *T. spiralis* ML, which indicated EuNPs-PAW-ES ICS may be more suitable for on-site detection of *T. spiralis* infection.

To confirm the feasibility of method, 1032 serum samples and 1032 pork samples from the field of china were detected by ICS and artificial digestion, respectively. Interestingly, we found that three serum samples were diagnosed as antibody positive by ICS, but only one pork sample was diagnosed as positive by artificial digestion, which may be due to the low sensitivity of artificial digestion in case of light *Trichinella* infection [37, 38]. But, analysis of the whole results, the result of ICS is highly consistent with artificial digestion, which indicate ICS could perform detection of anti-*Trichinella* antibody in endemic area.

In summary, ICS based on EuNPs-ES fluorescent probe was development for a novel detection method, which allows for sensitive, rapid and low-cost detection of *T. spiralis* infection. EuNPs-PAW-ES ICS showed the advantages in detecting of early infection, which can be used in excluding the false negative results in early infection. These results indicated that the EuNPs-ICS were successfully established and could help inspector more efficiently screening of sera and monitoring the early *T. spiralis* infection. Furthermore, the reaction condition ICS need to be optimized, including the performance of blood and plasma.

## Supplementary Information

**Additional file 1.** **Analysis of ML-ES and PAW-ES by SDS-PAGE.** A large number of secretion protein in the ML and PAW stage are shown in picture, respective. The results indicated that the two ES antigens can perform the next experiment.

**Additional file 2.** **Serum samples from pigs infected with 100 ML detected by Qiagen ELISA. **(S/*P* values were expressed as the means ± SD of five independent pigs).

**Additional file 3.** **Clinical serum samples detected by ICS.**

**Additional file 4.** **Larval densities in muscles of pigs infected with different doses of *T. spiralis *at 120 dpi.** By the digestion method, we took 50–100 g six parts of muscle tissues (tongue, shoulder, foreleg, diaphragm, gluteus and hind leg) from experimental pig to calculate the average lpg. This data obtained previous work in our laboratory, and had been published.

## Data Availability

All data generated or analyzed during this study are included in this published article.

## Abbreviations

EuNPs: Eu (III) nanoparticles
ELISA: Enzyme-linked immunosorbent assay
ML: Muscle larvae
PAW: Preadult worm
dpi: Days post-infection
LPG: Larvae per gram
ICS: Immunochromatographic strip
