# Supplementary figures and images for: Development of a rapid and sensitive immunochromatographic strip based on EuNPs-ES fluorescent probe for the detection of early Trichinella spiralis-specific IgG antibody in pigs
Source: Vet Res. 2021 Jun 11;52:85. doi: 10.1186/s13567-021-00951-9 (PMC8196438; doi:10.1186/s13567-021-00951-9)

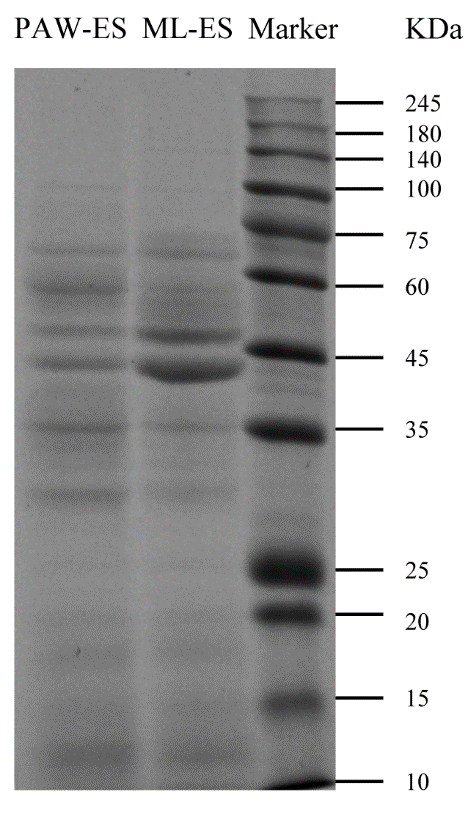

Supplement: Supplementary file 1 — Additional file 1. Analysis of ML-ES and PAW-ES by SDS-PAGE. A large number of secretion protein in the ML and PAW stage are shown in picture, respective. The results indicated that the two ES antigens can perform the next experiment. [file 13567_2021_951_MOESM1_ESM.docx]

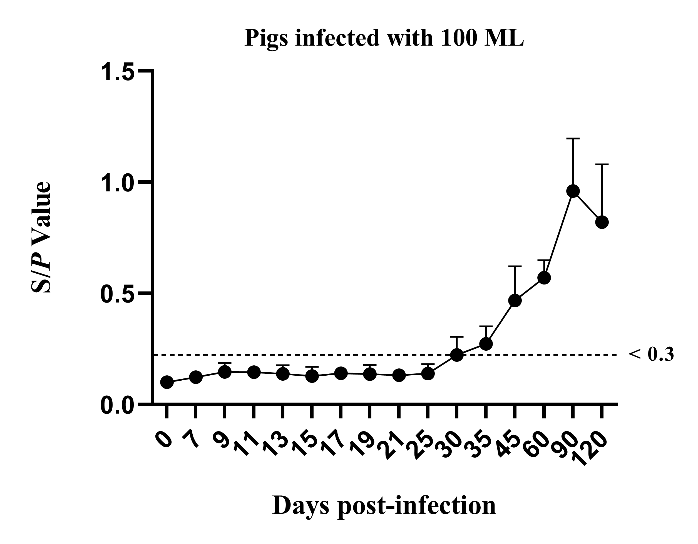

Supplement: Supplementary file 2 — Additional file 2. Serum samples from pigs infected with 100 ML detected by Qiagen ELISA. (S/P values were expressed as the means ± SD of five independent pigs). [file 13567_2021_951_MOESM2_ESM.docx]

**Positive**

**Negative**

**
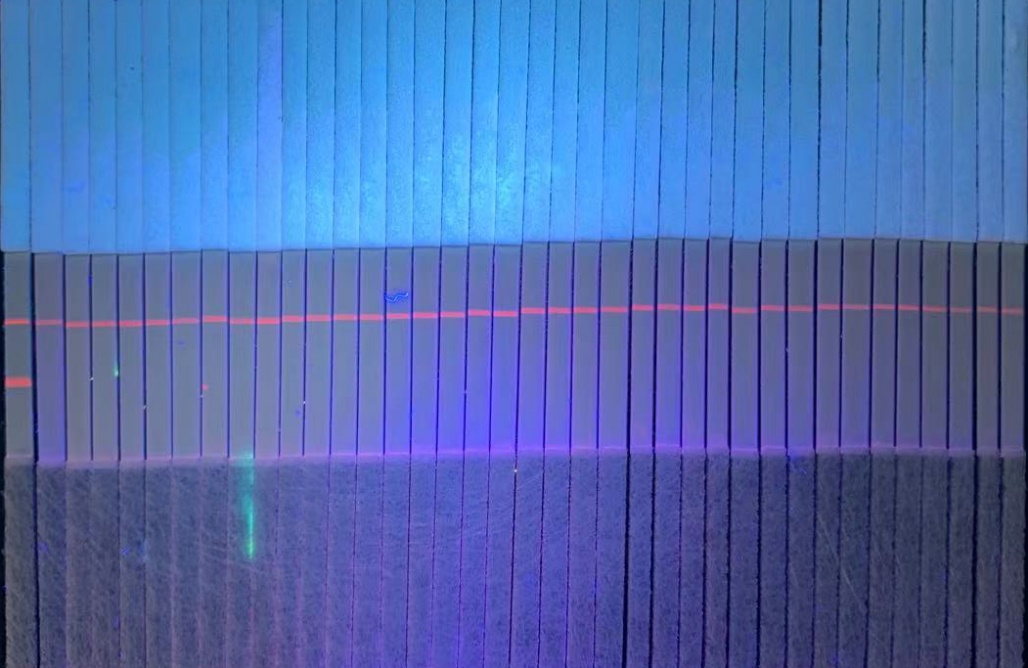
**

**Control line**

**Test line**

Supplement: Supplementary file 3 — Additional file 3. Clinical serum samples detected by ICS. [file 13567_2021_951_MOESM3_ESM.docx]
