# Supplementary material for: Development of a rapid and sensitive immunochromatographic strip based on EuNPs-ES fluorescent probe for the detection of early Trichinella spiralis-specific IgG antibody in pigs
Source: Vet Res. 2021 Jun 11;52:85. doi: 10.1186/s13567-021-00951-9 (PMC8196438; doi:10.1186/s13567-021-00951-9)
Supplement: Supplementary file 4 — Additional file 4. Larval densities in muscles of pigs infected with different doses of T. spiralis at 120 dpi. By the digestion method, we took 50–100 g six parts of muscle tissues (tongue, shoulder, foreleg, diaphragm, gluteus and hind leg) from experimental pig to calculate the average lpg. This data obtained previous work in our laboratory, and had been published. [file 13567_2021_951_MOESM4_ESM.docx]

| Lpg^a^ | | | | | | | | | | |
| --- | --- | --- | --- | --- | --- | --- | --- | --- | --- | --- |
| Doses^b^/location^c^ | Tongue | Shoulder | | Flexor | | Diaphragm | | Gluteus | Gastrocnemius | mean |
| 100 | 0.01 | | 0.003 | | 0.00 | | 0.03 | 0.00 | 0.00 | 0.007 |
| 1000 | 85.49 | | 13.63 | | 10.95 | | 46.70 | 4.98 | 8.03 | 28.30 |
| 10 000 | 533.11 | | 157.07 | | 87.85 | | 420.86 | 85.90 | 72.27 | 226.18 |

^a^Lpg: larvae per gram of muscle tissue.

^b^Doses: larval inoculation dose in pigs.

^c^location: muscle tissues from infected pigs.
